# CUL4B increases platinum‐based drug resistance in colorectal cancer through EMT: A study in its mechanism

**DOI:** 10.1111/jcmm.17585

**Published:** 2022-11-16

**Authors:** Jian‐Wu Luo, Chun‐Ming Wang, Jian‐Wei Su, Ting‐Zhuang Yi, Shao‐Hui Tang

**Affiliations:** ^1^ Department of Gastroenterology, The First Affiliated Hospital Jinan University Guangzhou China; ^2^ Guangxi Huiren Medical Technology Co., Ltd Nanning China; ^3^ Department of General Surgery The Second Affiliated Hospital of Guangxi Medical University Nanning China; ^4^ Gastrointestinal Medicine Affiliated Hospital of YouJiang Medical University For Nationalities Baise China; ^5^ Department of Oncology Affiliated Hospital of YouJiang Medical University For Nationalities Baise China

**Keywords:** colorectal cancer, CUL4B, drug resistance, EMT

## Abstract

Platinum‐based chemotherapy drugs play a very important role in the treatment of patients with advanced colorectal cancer, but the drug resistance of platinum‐based chemotherapy drugs is an important topic that puzzles us. If we can find mechanisms of resistance, it will be revolutionary for us. We analysed the differential genes, core genes and their enrichment pathways in platinum‐resistant and non‐resistant patients through a public database. Platinum‐resistant cell lines were cultured in vitro for in vitro colony and Transwell analysis. Tumorigenesis analysis of nude mice in vivo. Verify the function of core genes. Through differential gene and enrichment analysis, we found that CUL4B was the main factor affecting platinum drug resistance and EMT. Our hypothesis was further verified by in vitro drug‐resistant and wild‐type cell lines and in vivo tumorigenesis analysis of nude mice. CUL4B leads to platinum drug resistance in colorectal cancer by affecting tumour EMT.

## 1 INTRODUCTION

Colorectal cancer (CRC) is globally the third most common and the second deadliest tumour in humans regardless of gender.^1^ Some of its fatality can be attributed to its strong ties to genetics and environmental effects. In the past years, incidence and mortality of CRC has been steadily decreasing, partly accreditable to the health‐conscious society's increased tumour surveillance and better treatment.^2^ The typical CRC begins as an adenomatous polyp in the normal colonic epithelium as it accumulates mutations in known targetable oncogenes, tumour suppressor genes, and genes related to DNA repair.^3^ CRC is subclassified into stage 0, stage I, stage II, stage III, and stage IV depending on its depth of invasion, lymph node involvement, and metastasis, with stage 0 being the earliest, while stage IV signifying the most advanced. The prognosis of CRC is directly related to its staging, as well as the treatment available. As a rule of thumb, stage 0 is curable through operation, stage II requires a combination of adjuvant chemotherapy or radiotherapy along with a wider surgical resection to improve the success rate of treatment.^4^ Chemotherapy plays a pivotal role in CRC treatment regimens, specifically platinum‐based chemotherapeutic agents.^5^ Presently, there are many studies on the drug resistance in these platinum‐based agents for CRC, including fields such as DNA repair, tumour stem cells and epithelial‐mesenchymal transition (EMT), tumour microenvironment, etc.^6^

At this point in time, EMT is the importance research hotspot, hence worthy of exploration. During the EMT process, epithelial cells lose their apex‐basal polarity and cell connection, reorganize their cytoskeleton, and regulate the signalling pathways responsible for cell shape and movement. Concurrently, while the epithelial marker E‐cadherin is downregulated, the mesenchymal markers (such as vimentin, fibronectin) are upregulated. Not only that, cell migration and invasion also are increased, secretion of degrading enzymes are increased, and potential extracellular matrix is digested along the process.^7^ There are many studies that have proved that EMT is closely related to drug resistance in tumour.^8^ Paola et al. found that Gata6 made tumours easier to be killed by chemotherapy drugs by inhibiting EMT.^9^ However, how is EMT connected to the development of drug resistance of platinum‐based chemotherapeutics in tumour? In order to provide solid proof for future clinical decisions, we analysed the differential genes and enrichment status in recurrent and non‐recurrent patients who used platinum‐based drugs in order to identify the mechanism of platinum‐based drug resistance in CRC. And we found CUL4B increases platinum‐based drug resistance in colorectal cancer through EMT. The genes encoding a family of genes in this family of proteins form a complex that acts as an intracellular specific linker substrate for enzymatic activity, with a RING‐finger protein polygenic protein.^10^ The staining components of several regulators (including quality permitting DNA replication factor 1 in e).^11^

## 2 MATERIALS AND METHODS

### 2.1 Cell culture and managements

Cell culture and lentivirus packaging was completed as described in previous study.^12^ SW480 and HCT116 cells were purchased from the American Type Culture Collection (ATCC, Rockville, MD). Lipofectamine 2000 (Life Technologies, California, USA) was used for shRNA transfection according to the manufacturer's instructions. shRNA‐targeting CUL4B (shCUL4B) and negative control shRNA (shNC) were purchased from GeneChem. The lentivirus shCUL4B and its control were prepared in HEK293T. The infection was completed as described in previous study.^9^

Establish a stable oxaliplatin‐resistant colon cancer cell line as described in previous study.^13^

### 2.2 Colony formation assay

A colony formation assay was performed as described in previous study.^12^ After incubation at 37°C for 10–14 days, the cells were fixed with methanol and stained with Giemsa. The number of colonies containing more than 50 cells were recorded. All experiments were performed in triplicate, and the final statistical results were based on the number of colonies formed per well.

### 2.3 Transwell assay

The Transwell assay was performed as described in the previous study.^12^ For the cell invasion assay, Matrigel was diluted to a 5 mg/mL serum‐free medium and applied to the polycarbonate membrane filter of the reaction chamber at 37°C for 1 hour. 10 × 10^4^ cells suspended in 200 μl serum‐free were seeded into the invasion chamber. For migration assays, the process was similar, with 5 × 10^4^ cells spread on the top chamber, except without matrigel. All experiments were repeated three times, and the results were expressed as the percentage of cells that passed through the membrane.

### 2.4 Western blot

Western blot was performed as described in previous study.^14^ Afterwards, the membrane was incubated with the enzyme‐labelled secondary antibody at room temperature for 1 hour, and the ECL kit (Thermo Scientific, Rockford, IL, USA) was used for detection. Antibodies used: rabbit anti‐CUL4B (Proteintech, 12,916‐1‐AP), rabbit anti‐E‐Cadherin (Proteintech, 20,874‐1‐AP), rabbit anti‐Vimentin (Proteintech, 10,366‐1‐AP), and mouse anti‐GAPDH (Proteintech, 60,004‐1‐Ig). For proteins with significantly different molecular weights, PVDF membranes are tailored to incubate different antibodies.

### 2.5 Immunohistochemical staining

Immunohistochemical staining was completed as described in the previous study.^15^ The antibody is rabbit anti‐CUL4B antibody (dilution 1:1000), rabbit anti‐VIM antibody (dilution 1:1000) and rabbit anti‐ECAD antibody (dilution 1:1000). SP‐9000 kit (ZSGB‐BIO, Beijing, China) and DAB detection kit (ZSGB‐BIO) was used for staining according to the manufacturer's instructions. The expression of CUL4B, ECAD and VIM was quantitatively analysed by their staining intensity and positive rate. Staining intensity score and the positive rate score were multiplied to calculate the total expression of CUL4B and VIM: final value greater than 6 is considered high expression.

### 2.6 Tumour xenografts

In vivo tests were carried out as described in the previous study.^8^ Twelve male BALB/c nude mice aged 4–6 weeks were selected and randomly divided into two groups: control group and CUL4B knockdown group. Starting from Day 4, the mice from the CUL4B knockdown group were injected with oxaliplatin 5 mg/kg intraperitoneally every three days, as described in a previous study.^13^ The control group mice were intraperitoneally injected with the same amount of DMSO instead. Animal handling and experimental procedures were approved by the Ethics Committee of the first affiliated hospital of Jinan University.

### 2.7 Bioinformatic analysis

Bioinformatic analysis methods are as described.^15^ The transcriptome data and follow‐up data (recurrence post chemotherapy, etc) of CRC patients were obtained from the TCGA database (https://www.cancer.gov/about‐nci/organization/ccg/research/structural‐genomics/tcga).

Differential gene analysis was performed using ‘limma’ v3.28.14 of R software (https://www.bioconductor.org/packages/devel/bioc/vignettes/limma/inst/doc/users‐guide.pdf). Enrichment analysis was performed using the GO database (DAVID 6.8; https://david.ncifcrf.gov/). Gene co‐expression network was constructed through WGCNA package in R software using the gene expression data.^16^ The prognostic correlation and gene correlation analysis of TCGA patients are statistically analysed by GEPIA (Gene Expression Profiling Interactive Analysis) (http://gepia.cancer‐pku.cn/detail.php?gene). PPI: Hub genes are highly interconnected with nodes in the module and are considered to have important functions. The top 30 hub genes in the module network were selected as candidate genes for further analysis and validation. The STRING dataset (https://string‐db.org/) is an online biological resource that decodes interactions between proteins to obtain the true function of real proteins.^16^

### 2.8 Statistical analysis

The results were expressed as mean ± standard deviation (SD). All statistical analysis were completed via SPSS statistical software program (Version 22.0). The data were normally distributed, and the differences between groups were tested with Student's t‐test, two‐way analysis of variance was used instead if the variance was not uniform. The difference in survival was analysed through log‐rank test of Kaplan–Meier analysis. *p* value of < .05 was considered statistically significant (*p < .05, **p < .01, ***p < .001).

## 3 RESULTS

### 3.1 CUL4B is the core gene affecting oxaliplatin resistance in colorectal cancer

**FIGURE 1:**
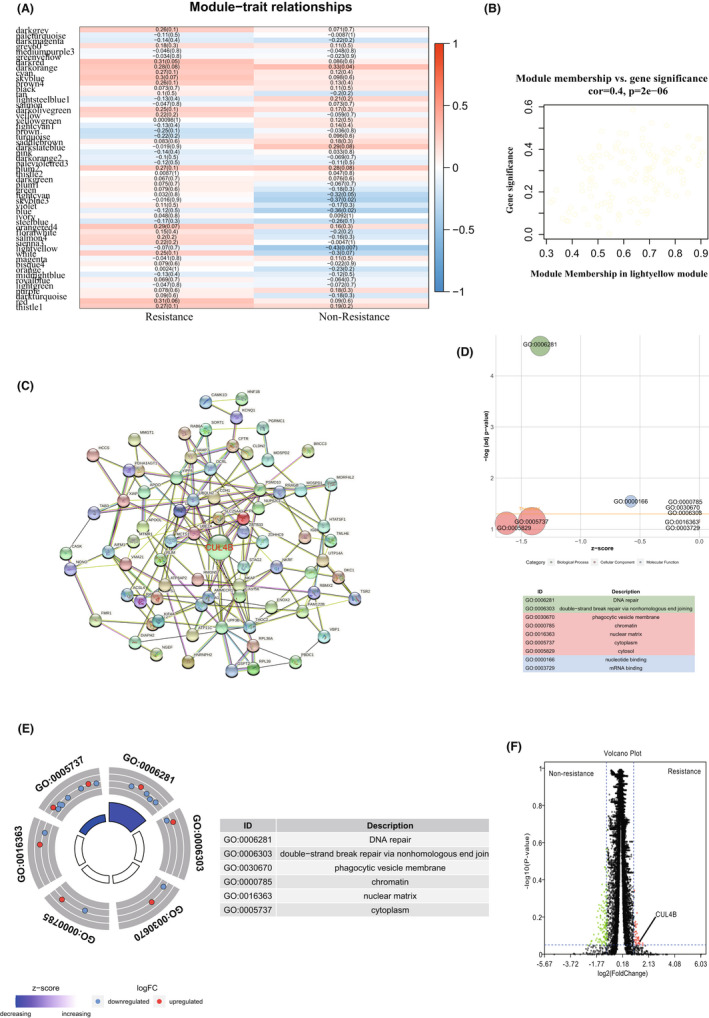
CUL4B is the core gene that affects oxaliplatin resistance in colorectal cancer. (A) Module‐trait relationships between platinum‐based drug resistance and non‐drug resistance. (B) Module Membership in ‘lightyellow’ module. (C) ‘lightyellow’ module protein interaction network. (D) Bubble chart of enrichment analysis of platinum‐based drug resistance and non‐drug resistance. The bubble size is related to the enrichment correlation. (E) Distribution chart of enrichment analysis of platinum‐based drug resistance and non‐drug resistance. The size of the loop is related to the enrichment correlation. (F) Volcano plot of the differential gene analysis of platinum‐based drug resistance and non‐drug resistance. Red dots are related to resistance genes

**FIGURE 2:**
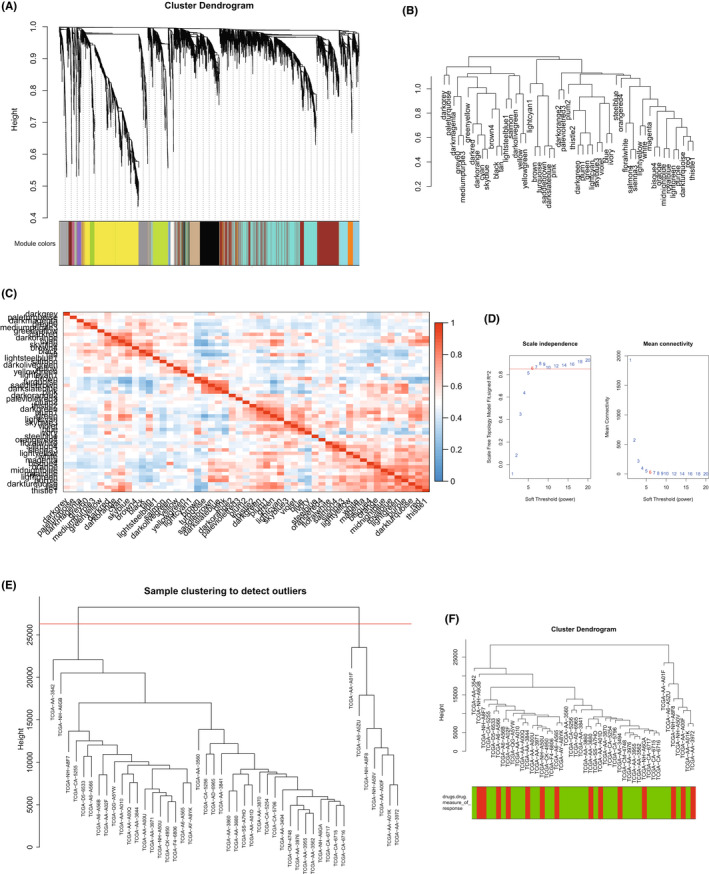
WGCNA analysis and verification of platinum‐based drug resistance and non‐drug resistance. (A) Cluster dendrogram with platinum‐based drug resistance and non‐drug resistance. (B) Dendrogram of platinum‐based drug resistance and non‐drug resistance. (C) Correlation analysis diagram of each module of platinum‐based drug resistance and non‐drug resistance. (D) Scale independence graph of platinum‐based drug resistance and non‐drug resistance. Six cut‐off points are in the diagram. (E) Sample clustering of platinum‐based drug resistance and non‐drug resistance to detect outlier. (F) Cluster dendrogram of platinum‐based drug resistance and non‐drug resistance

Through WGCNA R package, we analysed oxaliplatin tolerant and non‐tolerant patients in the TCGA database and found that the ‘lightyellow’ module is an important computing core module that affects oxaliplatin tolerance through the Module‐trait relationships method (Figure 1A). A comparison of Module membership vs. gene significance found that the ‘lightyellow’ module is indeed the core gene expression module that affects oxaliplatin tolerance (Figure 1B). Through the relevant verification as shown in Figure 2, it is found that our calculation model is indeed scientific.

In order to further seek the most important core genes in the ‘lightyellow’ module, we used the string database method. We found that CUL4B is in fact the core gene that affects the ‘lightyellow’ module (Figure 1C). Through enrichment analysis, we studied the potential pathways related to oxaliplatin tolerance, and found that DNA repair‐related pathways are significantly related to oxaliplatin tolerance (Figure 1D, E), and the expression of CUL4B in oxaliplatin tolerant group was significantly increased (Figure 1F). The WGCNA process is shown in Figure 2A–F.

### 3.2 CUL4B expression is associated with pathways such as EMT and drug resistance

**FIGURE 3:**
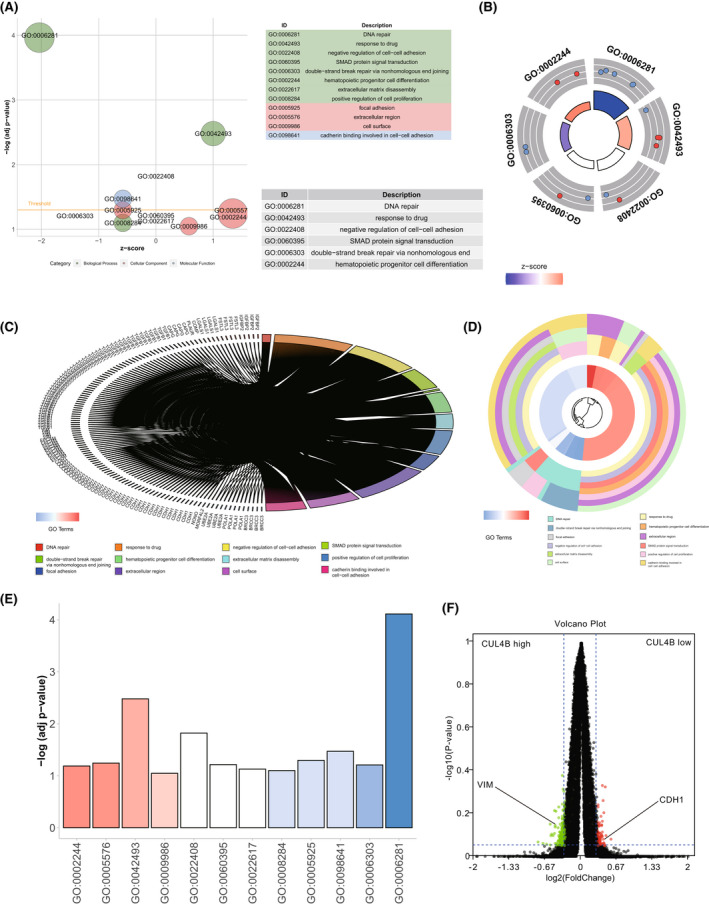
CUL4B expression is related to pathways such as EMT and drug resistance. (A) Bubble chart of enrichment analysis of high vs low CUL4B expression. The bubble size is related to the enrichment correlation. (B) The enrichment analysis distribution chart between high and low CUL4B expression. The size of the loop is related to the enrichment correlation. (C) Gene differential expression analysis of high and low CUL4B expression. (D) Functional enrichment analysis of high and low CUL4B expression. (E) Histogram of degree of enrichment. (F) The volcano plot of differential gene expression analysis of high and low CUL4B expression. The red dot is associated with low CUL4B expression. The green dot is associated with high CUL4B expression

In order to study the function of CUL4B, we analysed the pathway of CUL4B expression in the TCGA database through enrichment analysis, and found that high expression of CUL4B was not only related to the chemotherapy resistance‐related pathways (DNA repair, response to drug, double‐strand break repair via nonhomologous end joining), it was also significantly related to EMT‐related pathways (negative regulation of cell–cell adhesion, SMAD protein signal transduction, extracellular matrix disassembly, focal adhesion, cadherin binding involved in cell–cell adhesion) (Figure 3A,B). When we explored the correlation between genes and pathways, we found that genes related to CUL4B expression, such as CDH1 and VIM, are important genes that affect these pathways (Figure 3C–E). When the expression of CUL4B and EMT‐related genes were analysed, it was found that the expression of CDH1 and VIM was correlated with the expression of CUL4B (Figure 3F).

### 3.3 CUL4B expression is closely related to tumour development and progression

**FIGURE 4:**
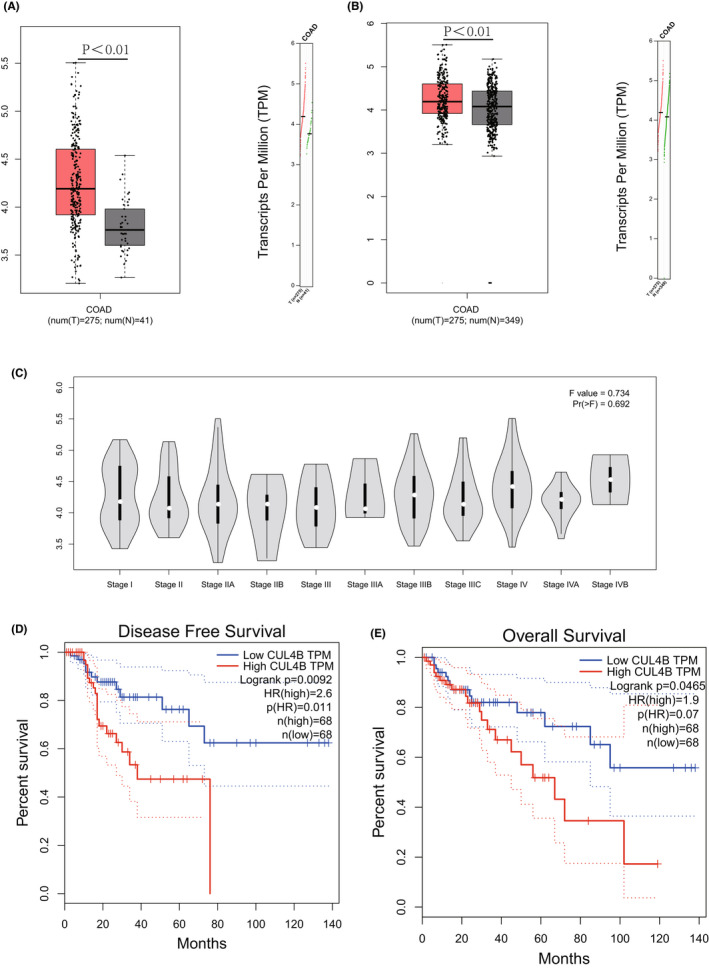
The expression of CUL4B is closely related to tumour development and progression. (A) The expression of CUL4B in cancer tissue obtained from TCGA database is significantly higher than that of normal tissues adjacent to tumour. (B) The expression of CUL4B in cancer tumour. (C) CUL4B expression in different stages of colorectal cancer obtained from the TCGA database. (D) Comparison of recurrence and survival time of colorectal cancer patients and their CUL4B expression. (E) Comparison of overall survival time of colorectal cancer patients and their CUL4B expression

In order to study the relationship of CUL4B in the development of CRC, we analysed the difference in expression of CUL4B between tumour tissue and normal tissue adjacent to the tumour (NAT) in TCGA, and found that the expression of CUL4B in tumours was significantly higher than that of its NAT (Figure 4A). By additionally comparing normal colorectal tissues and CRC tissues, it was also found that the expression of CUL4B was significantly higher in the cancer tissues (Figure 4B). A further study of relationship between CUL4B and tumour staging found that the expression of CUL4B was significantly increased in advanced gastric cancer (Figure 4C). Next, we analysed the survival and prognosis of tumour patients against CUL4B expression, and we found that the expression of CUL4B was closely related to recurrence and death of the patient (Figure 4D, E), indicating a huge impact.

### 3.4 In vitro experiments prove that CUL4B is an important factor in oxaliplatin resistance

**FIGURE 5:**
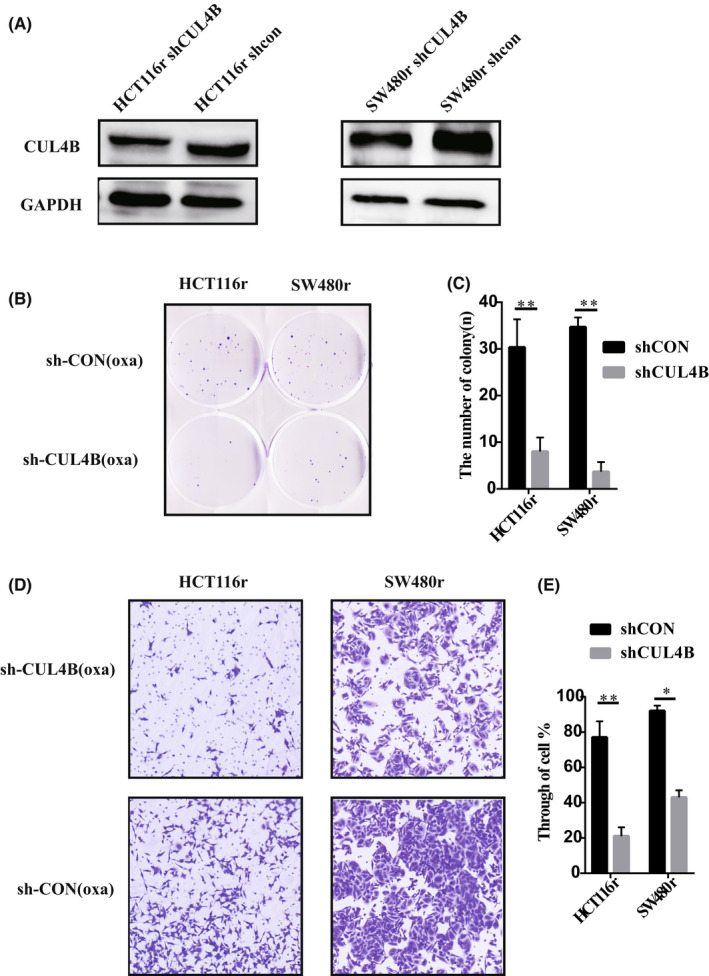
CUL4B function verified by drug‐resistant cell lines in vitro. (A) WB verified the knockdown effect of our drug‐resistant cell line. (B, C) proliferation of drug‐resistant cell lines with knockdown CUL4B was verified by cell colony test. (D, E) the invasion of CUL4B resistant cell lines was verified by cell Transwell test. *p < .05. **p < .01

After successfully constructing the CRC drug‐resistant cell lines HCT116r and SW480r in vitro, we knocked down CUL4B using lentivirus (Figure 5A). We conducted colony formation assay after applying oxaliplatin on both knockdown groups and the normal control groups, and found that after CUL4B knockdown, the resistance of drug‐resistant cell lines to oxaliplatin was significantly reduced (Figure 5B, C). Furthermore, by studying the effect of CUL4B on cell invasion of drug‐resistant cell lines, we found that knocking down CUL4B can significantly reduce the invasion ability of drug‐resistant tumour cells (Figure 5D, E), indicating that CUL4B is an important cause of oxaliplatin resistance.

### 3.5 In vivo experiments found that CUL4B knockdown can affect the effect of oxaliplatin on tumour volume and proliferation

**FIGURE 6:**
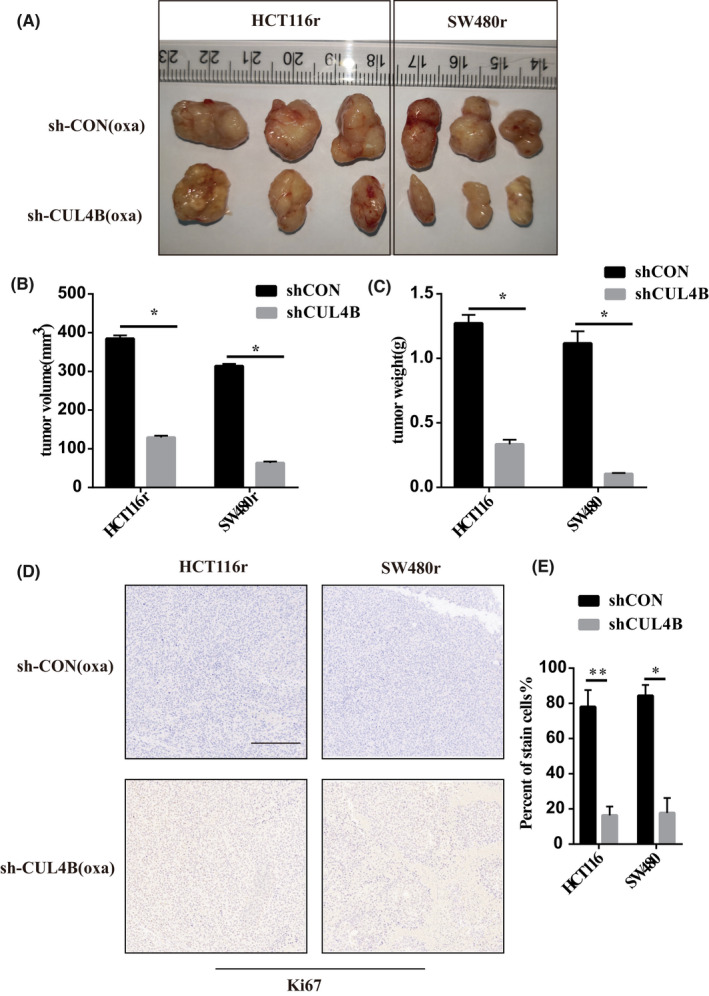
CUL4B function was verified by subcutaneous tumour model of drug‐resistant cell lines in vivo. (A, B and C) After CUL4B knockdown by the resistance to oxaliplatin decreased significantly. (D, E) oxaliplatin can significantly inhibit the proliferation and growth of tumour cells after CUL4B knockdown. *p < .05. **p < .01. The ruler is 200 um in length

After proving the importance of CUL4B in oxaliplatin resistance in vitro, we then performed subcutaneous tumour formation experiments in nude mice. We subcutaneously injected CUL4B knockdown cells and control drug‐resistant cells into nude mice, and later intraperitoneally injected oxaliplatin into nude mice on the fourth day. Finally, by comparing the tumour size and weight, we found that the tumour volume in the CUL4B knockdown group was significantly higher than that of the control (Figure 6A–C). Through immunohistochemistry, we found that the proliferation ability of the CUL4B knockdown group was significantly lower than that of the control (Figure 6D, E). It shows that CUL4B in vivo can indeed affect the occurrence of oxaliplatin resistance in tumours.

### 3.6 CUL4B affects drug resistance by affecting the occurrence and development of tumour EMT

**FIGURE 7:**
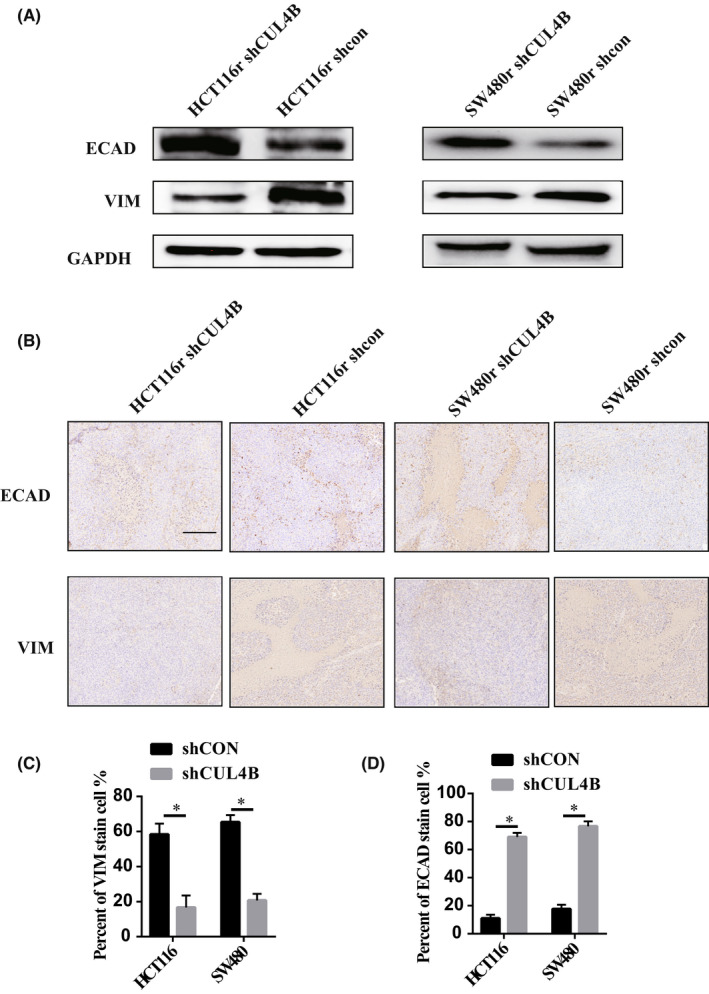
EMT was significantly inhibited after CUL4B was knocked down. (A) after CUL4B knockdown, the expression level of ECAD in drug‐resistant cells increased and VIM expression was down‐regulated. (B, C, D) After knockdown CUL4B, the expression of ECAD increased and VIM down‐regulated in nude mice. *,*p* < .05. The ruler is 200 um in length

The studies above have shown that CUL4B can regulate the occurrence of chemotherapeutic drug resistance, especially those of oxaliplatin, by affecting the occurrence of EMT. In order to verify in vivo and in vitro, we compared the EMT‐related markers ECAD and VIM between CUL4B knockdown and control variants of drug‐resistant strains and found that after knocking down CUL4B, mesenchymal cells decreased while epithelial cells increased (Figure 7A). In vivo tumour formation assay also found that after CUL4B knockdown, the tumour was composed of significantly more epithelial cells than mesenchymal cells (Figure 7B–D), showing that CUL4B can affect EMT and thereby affect drug resistance.

## 4 DISCUSSION

The Cullin4 (CUL4) family has two members, namely CUL4A and CUL4B. These 2 proteins have a high degree of homology of 83%.^17^ In recent years, the role of CUL4B in solid tumours has been gradually uncovered and thus attracted widespread attention in our scientific community. Among them, many studies have shown that CUL4B is abnormally expressed in a variety of diseases and physiological processes.^18^ These studies have clearly demonstrated the various roles of CUL4B in cell proliferation, DNA damage and repair, cell cycle progression, cancer metastasis and invasion, DNA methylation and histone acetylation modification, and signalling pathways.^19^ Our study has for the first time found that CUL4B is an important target gene for resistance of platinum‐based drugs in colorectal cancer. Through in vitro and in vivo experiments, we revealed that CUL4B influences the occurrence of EMT and regulates the progression of platinum‐based chemotherapeutic resistance in CRC and is of great significance to the survival and prognosis of these patients.

Epithelial–Mesenchymal Transition (EMT) is a process in which epithelial cell loses its apical‐basal polarity and cell–cell adhesion, and transition into the more aggressive mesenchymal cell. On the contrary, Mesenchymal‐Epithelial Transition (MET) is its reverse. MET includes the transformation of cells from a motile, multipolar mesenchymal type to a polarized epithelial type. EMT is involved in many biological and pathological processes, its vital in embryonic development, wound healing, cancer cell metastasis and drug resistance.^20^ The link between EMT and cancer cell drug resistance has been suggested since the early 1990s. Sommers et al. found signs of EMT in two MCF‐7 cell lines resistant to doxorubicin and one ZR‐75‐B cell line resistant to vinblastine.^21^ More and more researchers realize that many cancers like pancreatic cancer, bladder cancer, breast cancer and others, drug resistance is often accompanied by EMT.^22^ One of the main function of CUL4B is to affect the DNA repair process.^23^ The latest study has found that CUL4B also plays an important part in the occurrence and development of EMT, with several distinct studies finding CUL4B regulating EMT via multiple pathways.^24^, ^25^, ^26^ EMT seems unlikely to malfunction and appears to be an important component, but MT can present all its difficulties in a tumour‐like manner to a large number of anticancer drugs.^27^ Important histological characteristics of different types of genes, different types of genes, such as other cancer drug clinical studies may be possible if the expression or histological characteristics of tumours are measured.^28^ However, there was no research on the effect of CUL4B on platinum‐based chemotherapeutic drug resistance. Our research has filled in that gap of knowledge and has found that CUL4B can affect the development of platinum‐based drug resistance through regulation of EMT.

In summary, through our current study, we found that CUL4B can affect the progression and prognosis of colorectal cancer by playing a vital role in oxaliplatin resistance. In the follow‐up study, we designed in vivo and in vitro experiments and found that CUL4B can regulate the occurrence and development of oxaliplatin resistance in CRC by affecting the EMT in CRC.

## AUTHOR CONTRIBUTIONS

**Jian‐Wu Luo:** Data curation (equal); investigation (equal); methodology (equal). **Chun‐Ming Wang:** Conceptualization (equal); data curation (equal). **Jian‐Wei Su:** Conceptualization (equal); resources (equal); software (equal). **Ting‐Zhuang Yi:** Conceptualization (equal); investigation (equal); project administration (equal). **Shao‐Hui Tang:** Conceptualization (equal); investigation (equal); project administration (equal); visualization (equal); writing – original draft (equal).

## CONFLICT OF INTEREST

The authors declare no competing interests.

## Data Availability

The data that support the findings of this study are available on request from the corresponding author.
